# Estimating the Severity of Oral Lesions Via Analysis of Cone Beam Computed Tomography Reports: A Proposed Deep Learning Model

**DOI:** 10.1016/j.identj.2024.06.015

**Published:** 2024-07-26

**Authors:** Sare Mahdavifar, Seyed Mostafa Fakhrahmad, Elham Ansarifard

**Affiliations:** a Dept. of Computer Science and Engineering and IT, Shiraz University, Shiraz, Iran; b Dept. of Prosthodontics, School of Dentistry, Shiraz University of Medical Sciences, Shiraz, Iran; c Biomaterials Research Center, School of Dentistry, Shiraz University of Medical Sciences, Shiraz, Iran

**Keywords:** CBCT image, Radiology report, Oral lesions, Deep learning, Text classification, Machine learning

## Abstract

**Objectives:**

Several factors such as unavailability of specialists, dental phobia, and financial difficulties may lead to a delay between receiving an oral radiology report and consulting a dentist. The primary aim of this study was to distinguish between high-risk and low-risk oral lesions according to the radiologist's reports of cone beam computed tomography (CBCT) images. Such a facility may be employed by dentist or his/her assistant to make the patient aware of the severity and the grade of the oral lesion and referral for immediate treatment or other follow-up care.

**Methods:**

A total number of 1134 CBCT radiography reports owned by Shiraz University of Medical Sciences were collected. The severity level of each sample was specified by three experts, and an annotation was carried out accordingly. After preprocessing the data, a deep learning model, referred to as CNN-LSTM, was developed, which aims to detect the degree of severity of the problem based on analysis of the radiologist's report. Unlike traditional models which usually use a simple collection of words, the proposed deep model uses words embedded in dense vector representations, which empowers it to effectively capture semantic similarities.

**Results:**

The results indicated that the proposed model outperformed its counterparts in terms of precision, recall, and F1 criteria. This suggests its potential as a reliable tool for early estimation of the severity of oral lesions.

**Conclusions:**

This study shows the effectiveness of deep learning in the analysis of textual reports and accurately distinguishing between high-risk and low-risk lesions. Employing the proposed model which can Provide timely warnings about the need for follow-up and prompt treatment can shield the patient from the risks associated with delays.

**Clinical significance:**

Our collaboratively collected and expert-annotated dataset serves as a valuable resource for exploratory research. The results demonstrate the pivotal role of our deep learning model could play in assessing the severity of oral lesions in dental reports.

## Introduction

**Fig. 1:**
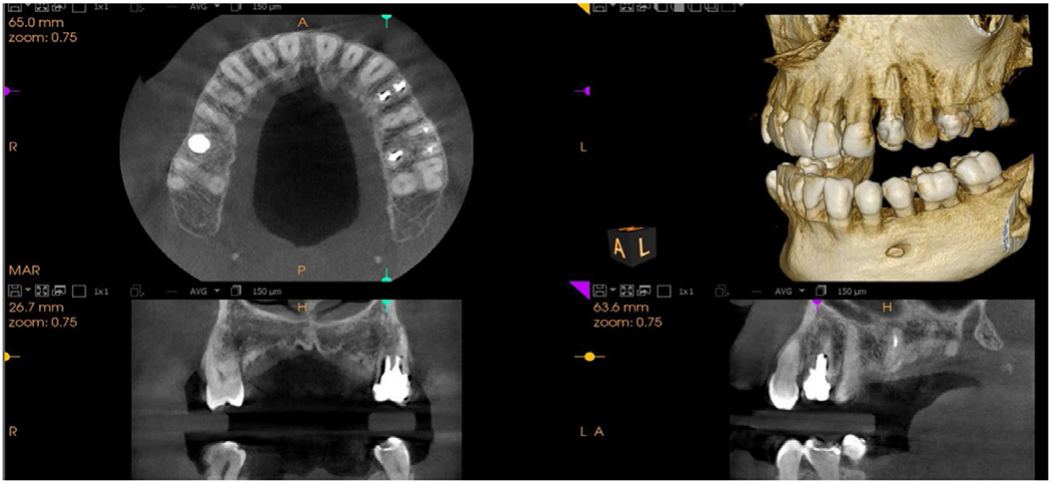
A typical CBCT image.

Cone beam computed tomography (CBCT) radiography is a type of dental imaging that captures three-dimensional images of the teeth, as well as all the bones and soft tissues within the mouth. Analysing three-dimensional CBCT images has become an indispensable procedure for the diagnosis and treatment planning of orthodontic patients.^1^ The applications of CBCT radiography are diverse, including informing attending physicians of hidden teeth, diagnosing joint disorders, precisely placing implants, examining the condition of the jaw, sinuses, and nerve canals, diagnosing tumours in the jaw and mouth, assessing bone structure, identifying the source and origin of pain, and guiding surgical interventions. As a result, CBCT imaging, unlike other methods, can reveal a broad spectrum of oral, jaw, and facial issues. A sample of CBCT images is shown in Figure 1.

Some of the issues revealed by CBCT images require urgent attention, such as the presence of malignant tumours in the mouth and jaw areas, necessitating swift treatment to prevent endangering the person's life. Another set of CBCT images includes those that show significant issues that require follow-up and treatment, but the urgency may not be as high, and a delay may not pose a serious risk periapical (radicular) and dentigerous cysts are examples of this group. The third situation arises when a problem that needs follow-ups and treatments is identified in CBCT images, but no emergency exists as an example for this situation, suppose that in the upper jaw of a given case, the bone depth near the nerve canals and sinus cavities is low. If the individual decides to implant in that area, a sinus lift would be necessary to create more depth and distance. However, since this is not considered a problem or a disease, and the person may choose not to undergo implantation, addressing this matter is not particularly urgent. Other examples of the third category of issues include lesions such as fibro-osseous dysplasia and Osteosclerosis. The fourth situation is when conditions are entirely normal, or an unimportant issue such as Stafne Bone Cavity (SBC) is detected which does not need any care or treatment. As another example, suppose a case in which an impacted tooth is not associated with vital structures (IAN or sinus) and there is no evidence of root resorption in adjacent teeth. In this case, no treatment for the impacted tooth is necessary.

As indicated by the illustrations above, the distinction among the four mentioned categories lies in the severity level of the patient's condition. Category 1 represents the most high-risk cases, whereas Category 4 denotes situations where everything is normal or where an unimportant finding exists. Definitions for these four categories are provided in Table 1. Given that each category encompasses various types of issues, Table 1 also offers examples for further clarification of the problems and lesions associated with each category.

**Table 1:** Description of different severity levels of oral problems according to CBCT radiography.

	Description (severity level)	Example of lesions
**Category1**	Lesions that threaten survival and require urgent treatments	Malignancies (eg, SCC, sarcoma, melanoma, adenocarcinoma), Some benign tumours (eg, ameloblastoma) Aggressive cysts (eg, OKC)
**Category2**	Important to follow up as soon as possible, but not survival-threatening	Supernumerary teeth associated with vital structures, inflammatory diseases (eg, periapical cysts), nonaggressive cysts (eg, dentigerous cysts)
**Category3**	Lesions that Need follow-ups and treatments whenever possible, but no emergency exists	Fibro-osseous dysplasia, osteosclerosis
**Category4**	No problem detected, or only unimportant issues exist	Impacted teeth having no association with vital structures, Stafne Bone cyst

CBCT, cone beam computed tomography.

Due to various factors such as patients’ lack of knowledge about their conditions, the unavailability of specialists, unfavourable financial conditions, work-related issues, or the fear associated with being in a medical environment (especially during a pandemic), a delay may exist between receiving a radiology report and consulting a doctor. In some cases, especially in the first category of the radiology report results, this time delay can pose significant and irreparable risks for the patient. In such situations, designing a system capable of accurately diagnosing the severity of the patient's problem by examining and analysing the radiology report becomes crucial. This acquired knowledge holds significance at various stages of a patient's life, particularly in aiding physicians in assessing a patient's health progress during treatment. The extraction of knowledge from these summaries enhances the evaluation of treatment quality, providing benefits to both patients and healthcare facilities.^2^, ^3^, ^4^ Providing timely warnings about the need for follow-up and prompt treatment can shield the patient from the risks associated with procrastination and delay. On the other hand, if there is no acute problem, informing the patient of this condition can help alleviate anxiety before their doctor's visit.

To achieve the stated goal, the approach of sentiment analysis and text classification can be employed. This approach allows the severity and seriousness of the problem to be reported based on the way the radiologist expresses the issue in the text. Sentiment analysis is a branch of natural language processing (NLP), which, in simple terms, involves examining the point of view and opinion expressed about a specific topic in a text.

Sentiment analysis techniques are designed to determine the expressed sentiment regarding a particular subject or the overall contextual polarity of a document. Emerging from the realm of web mining, the advancement of sentiment analysis approaches frequently focuses on analysing highly subjective texts, notably customer reviews.^5^, ^6^, ^7^ This operation finds applications in various fields. For instance, in the commercial sector, it can be used to analyse customer opinions about a particular product. Similarly, it has valuable applications in the field of medicine and health. There is a wealth of healthcare information available online, such as personal blogs, social media, and websites ranking medical topics, and extracting this information may not be straightforward to obtain. Therefore, sentiment analysis offers numerous benefits, including leveraging medical information to achieve optimal results for improving healthcare quality. Approaches in sentiment analysis can be categorized based on the techniques used, the structure of the dataset, the level of ranking, and other factors.

In recent years, sentiment analysis has made inroads into the realm of medicine. Leveraging the advances in big data, medical professionals are harnessing this opportunity to diagnose and analyse emotions using available data, ultimately improving medical services. This approach departs from traditional systems that rely on patient questionnaires for initial diagnosis. NLP enables the analysis of the vast patient dataset, which is then structured into a training dataset to educate an automated system. This research is aimed at distinguishing high-risk patients from low-risk patients and providing crucial assistance, particularly during challenging situations such as the Pandemic.

The rest of the paper is organized as follows. The ‘Material and methods’ section is devoted to material and methods. In this section, first, the process of dataset construction is illustrated. The dataset is a collection of CBCT radiography reports annotated by specialists. The proposed scheme for the classification of the reports and detection of the severity levels, which is a deep learning model is then described in detail. Evaluation results are presented in the ‘Results and evaluations’ section. A discussion is given in the ‘Results’ section. Finally, the ‘Discussion’ section concludes the paper.

## Material and methods

### Dataset construction

In this study, we used the dental radiology database provided by Shiraz University of Medical Sciences as our primary data source. From this extensive database, a total of 1134 patient records were carefully extracted to form the basis of our research. When gathering this set of data from the whole collection of the reports, in order to ensure the diversity of selection so as to accurately capture various clinical situations, the following points have been considered carefully:

- For each of the four severity levels described in the ‘Introduction’ section, a sufficient number of cases has been selected.
- Every type of usual oral problem and lesions, examples of which were given in Table 1, has been tried to be included in the final dataset so that comprehensive training for the classifiers can be carried out.

**Fig. 2:**
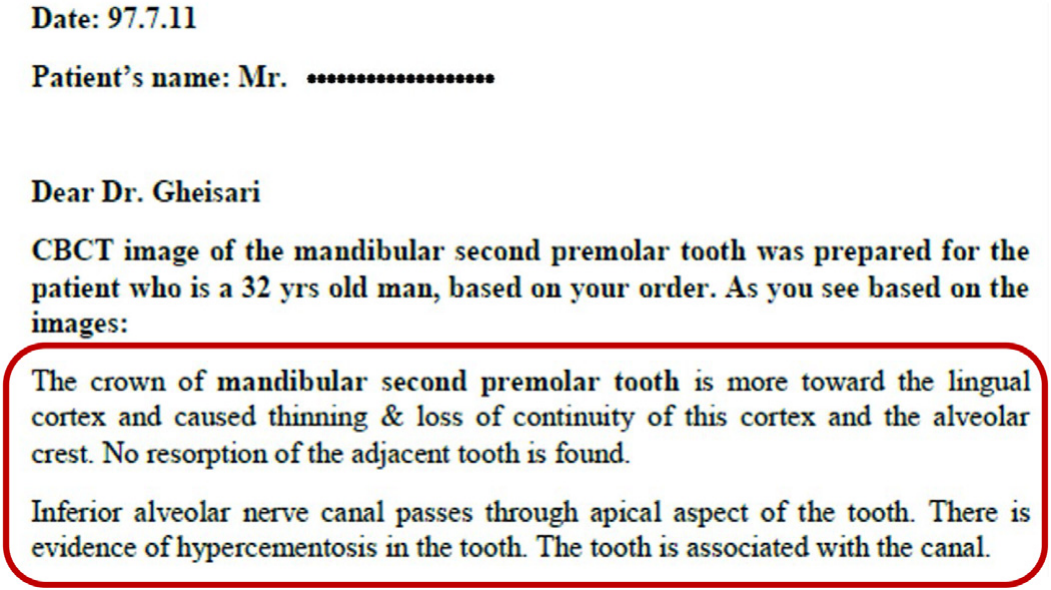
A sample of a radiologist's report for a CBCT image.

It's worth noting that clinical records often contain introductory information that does not contribute to the practical insights needed for our research and can be ignored. An example of the extracted CBCT reports is shown in Figure 2.

After the extraction of data samples, the major and most sensitive task was annotating the severity level of each sample (ie, one of the labels 1, 2, 3, or 4) according to the definitions given in the ‘Introduction’ section. This annotation process was managed by the third author of the manuscript, who is a prosthodontist, with the thoroughgoing cooperation of a maxillofacial radiologist as well as an oral and maxillofacial Medicine specialist. In order to have a reliable labelled dataset, cross-validation of assigned labels was performed by the mentioned specialists during the annotation process. Thereby, the severity level assigned to every sample was verified and confirmed by each of the specialists. However, there were a few cases of disagreement in determining the proper category label for some samples. This issue was resolved via further investigations and intraparty discussions carried out by the three specialists.

After finalizing the annotation process, the dataset of CBCT reports was organized into four distinct groups, each labelled as 1, 2, 3, or 4. Table 2 shows an example of each category with its assigned label. Notably, due to the variance in sample sizes among these groups, in the next section, we will employ a random oversampling technique to mitigate potential bias and ensure a balanced representation of data across all categories. This will be quite helpful in avoiding any bias of the classifiers (which will be trained using the given dataset) towards any specific class label.

**Table 2:** Example reports for different severity levels.

Radiologist's report	Label
CBCT image of the proposed area was prepared for the patient based on your order. As you see in the images: There is a well-defined mixed lesion with **thick sclerotic border** in left side of mandible which is extended from #n to #. The lesion caused considerable **expansion of lingual cortical plate** and thinning of this **cortex and buccally displacement of IAN.** Based on CBCT data, the most probable diagnoses are: **Ossifying fibroma, chondrosarcoma.**	1
CBCT image of the proposed area was prepared for the patient based on your order. As you see based on the images: The super **neumary tooth (SN)** is **oriented horizontally**. It is located palatally to #q tooth. It caused **displacement of tooth #q**, blunting of #q and **loss of continuity of palatal cortex**. SN has **involved the incisive foramen.**	2
CBCT of the #, tooth was prepared based on your order. As you see on the images: **The #, **tooth is horizontally oriented**. Direct contact (with preserving cortex) is noticed between the **IAN** canal and #. Thinning of **lingual cortical plate is evident.**	3
Thanks for your consult; CBCT of the mandible was performed for the patient based on your request. Measurements for **implant placement** were done for the patient and are presented on the CBCT sheets. Note: *There is an **enostosis-like bony structure** in the left side of the mandible in the molar region.	4

CBCT, cone beam computed tomography.

### Methodology

**Fig. 3:**
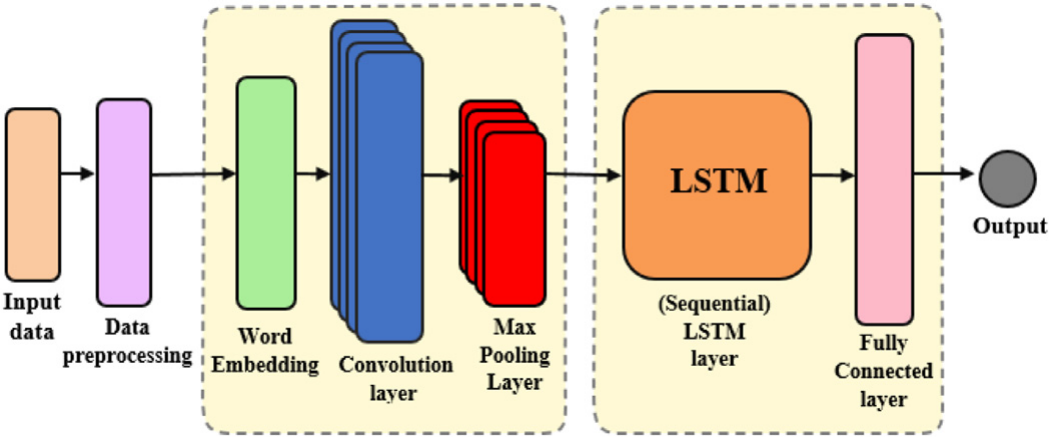
Structure of a CNN-LSTM model.

In this section, we present an overview of the proposed classification model which is a CNN_LSTM^1^ scheme, as depicted in Figure 3.

CNN is a specialized kind of feedforward neural network that is regularized to autonomously learn feature engineering through the optimization of filters. Its distinctive feature is the incorporation of an automatic feature extractor during training, eliminating the need for manual implementation of this crucial component.^8^ LSTM networks are considered among the most effective solutions for sequence prediction problems. The most advantageous property of LSTM is that it can remember patterns over long sequences. This gives LSTM an advantage over standard feedforward and recurrent neural networks, which lack the ability to use long-range observations for future predictions.^9^

Generally, deep learning methods are considered as suitable solutions for unstructured data, where a high level of abstraction is needed to extract features. One of the cases in which deep learning methods can be properly employed is for NLP tasks such as the present study, where there's a need to identify the complex relationships among the words and other elements of the text. More specifically, in CNN-LSTM, the CNN component is aimed to extract higher levels of word representation sequences and inputs them into the LSTM component to obtain sentence representations. This can capture the local features of the text and acquire the temporal semantics of the sentence, which results in effectiveness in emotional and sentiment classification.

As mentioned before, the collected data of the present study are classified into four classes based on the severity of the patient's conditions, labelled 1, 2, 3, and 4. The experiments were conducted on two datasets in both balanced and imbalanced modes: one with the dataset containing four categories, and the other with two categories. For the second case, the samples labelled 1 and 2 were considered as emergency cases and merged into a single class labelled 1. Similarly, the samples labelled 3 and 4 were merged into another class labelled 2 as nonemergency patients. We made this decision due to the proximity of the severity rates in categories 1 and 2, as well as categories 3 and 4. In this mode, the classification model is trained on a dataset with two categories. Finally, in order to have a balanced dataset and avoid biases towards some specific class labels, we utilized an oversampling technique, which equalized the number of samples among different classes, and increased the total number of data samples to 1416.

The proposed model performs three major steps, that is, data extraction and preprocessing, model training, and model testing, as will be described in the following subsections.

### Data extraction and preprocessing

Information extraction (IE) is an NLP technique that entails the automatic extraction of structured information from unstructured text. The objective of IE is to transform textual data into a more organized and usable format. This process usually includes identifying specific entities, relationships, and attributes within a given text. As depicted in Figure 2, each patient's record contains various information and attributes, of which only the text report of the CBCT image is required.

Data preprocessing involves preparing raw data before constructing model. This process involves the removal of symbols and numbers from the CBCT textual report, and preparing the dataset for training by the CNN-LSTM model. In this step, all clinical reports with various labels undergo preprocessing. The dataset may contain several undesirable symbols and numbers that require correction or removal to enhance the performance of the model. Therefore, this process entails the removal of all symbols and numbers. For example, for the given report in Table 2 which is labelled as Category 3, all valueless symbols such as ‘#’, ‘*’, ‘(‘, and ‘)’ were eliminated.

### Model training

Fetching the dataset from the file is followed by the extraction of all corresponding words (feature words) through word embedding, which represents words as vectors in a multidimensional space, capturing semantic relationships among them. These embeddings enhance the understanding of word meanings in NLP tasks, contributing to improved performance across various applications.

After vectorizing the textual data, in order to enhance training effectiveness, data shuffling is performed using a random seed. The shuffled dataset is then divided into 80% to 20% splits for training and testing, respectively. The training data is then given to the CNN-LSTM model, and the model is constructed, accordingly.

### Model testing

In this phase, the constructed CNN-LSTM model should be evaluated in the prediction of the class label for any unseen sample included in the test data, which constitutes 20% of the entire dataset. This step enables a comprehensive assessment of the model's effectiveness.

The process begins with data preprocessing, encompassing the removal of symbols and numerical values from every instance of the test dataset. Subsequently, the prepared dataset is fed into the pretrained model for predictive analysis.

## Results and evaluations

In this section, the evaluation criteria used for assessment of the proposed model, as well as the experimental results achieved for different cases are illustrated. A comprehensive investigation of the proposed model compared to other techniques is also carried out.

### Configuration and model parameters

The suggested CNN-LSTM model was implemented using the Keras library in the Python programming language. The training dataset was randomly divided into 32 batches per epoch, and the model was trained for 10 epochs with a learning rate of 0.01. Categorical cross-entropy was chosen as the loss function due to its suitability for multiclass classification problems, while accuracy was used as the validation metric. Adam was selected as the optimizer. The best hyper-parameters were identified using a grid search algorithm, with ranges derived from related work in the field. The predictions were compared and evaluated against the labels of the test dataset. Furthermore, accuracy, precision, recall, F1-score, and ROC-AUC metrics were computed during the evaluation.

### Evaluation metrics

We used different evaluation metrics to investigate the performance of models, including accuracy, precision, recall, F1-score, and ROC-AUC. All of these metrics are calculated based on the following parameters:

• True positives (TP): The count of reports with an expected label correctly classified.
• True negatives (TN): The count of reports with an unexpected label is accurately classified.
• False positives (FP): The count of reports incorrectly classified as expected results.
• False negatives (FN): The count of reports incorrectly classified as unexpected results.

**Accuracy** is described as the ratio of correct outcomes to the total cases evaluated, and it is applicable to both binary and multiclass classification challenges.

(1) $$Accuracy\;=\;\frac{TP+TN}{TP+FP+TN+FN}$$

**Precision** of a classification is defined by the proportion of accurately classified exact and expected observations out of all specific observations.

(2) $$Precision\;=\;\frac{TP}{TP+FP}$$

**Recall** is an assessment of the effectiveness in classifying positive observations. It is expressed as the ratio of TP to the total number of positive observations.

(3) $$Recall\;=\;\frac{TP}{TP+FN}$$

**F1-Score** combines the precision and recall scores of a model.

(4) $$F1\_Score\;=\;\frac{2\times Precision\times Recall}{Precision+Recall}$$

**AUC**^2^ is the area under the **ROC** curve^3^, which provides an aggregate measure of performance across all possible classification thresholds and evaluates how well a binary classification model can distinguish between the two classes.

## Results

In this section, we evaluate the effectiveness of the proposed CNN-LSTM model and compare it with other machine learning (ML) models, including MNB, LR, LSVC, RF, MLP, DT, and SVM, across both datasets in balanced and imbalanced modes. Specific parameters were employed in training the deep learning model, utilizing the Categorical Cross-Entropy loss function and the Adam algorithm for optimization. The neural networks underwent training for 10 epochs with a batch size of 32, and K-fold cross-validation (*k* = 5) was implemented to enhance the model's performance. Dataset splitting was carried out at an 80:20 ratio, allocating 80% for training the models and reserving 20% for evaluating their performance. Moreover, All ML models were trained using the transformed data through TF-IDF, with 80% of the data assigned to each fold. To optimize training effectiveness, data shuffling has been carried out using a random seed. The labelled datasets have been partitioned into 80% to 20% splits for training and testing, respectively. The training data is subsequently fed into a variety of models, encompassing MNB, LR, LSVC, RF, MLP, DT, and SVM, all operating at the document level.

Table 3 displays the outcomes of models on an imbalanced dataset containing four classes. LSVC exhibits superior performance compared to other ML methods, achieving an accuracy of 69.6%. CNN-LSTM outperforms all models, achieving the highest accuracy at 90.6%. Consequently, the overall performance of deep learning surpasses that of ML algorithms on the imbalanced dataset with four distinct classes.

**Table 3:** The performance of models for imbalanced datasets with four classes.

Classifiers	Accuracy
NB	59.8
LR	65.3
**LSVC**	**69.9**
MLP	66.0
SVM	64.7
DT	57.9
RF	63.3
**CNN-LSTM**	**90.6**

The results in bold indicate the highest accuracy and performance of the machine and deep learning models.

Table 4 presents the results of models on a balanced dataset comprising four classes. SVM demonstrates exceptional performance, outperforming other ML methods with an accuracy of 90.7%. CNN-LSTM surpasses all models, achieving the highest accuracy at 97.3%. Thus, the overall efficacy of deep learning exceeds that of ML algorithms on the balanced dataset with four distinct classes. Furthermore, the results indicate an improvement in the performance of all models in the balanced dataset.

**Table 4:** The performance of models for a balanced dataset with four classes.

Classifiers	Accuracy
NB	75.5
LR	84.0
LSVC	88.2
MLP	90.4
**SVM**	**90.7**
DT	85.5
RF	90.6
**CNN-LSTM**	**97.3**

The results in bold indicate the highest accuracy and performance of the machine and deep learning models.

Table 5 shows the results of model evaluations on an imbalanced dataset encompassing two classes. LSVC demonstrates outstanding performance, outshining other ML methods with accuracy, precision, and recall of 80.3%, an F1 of 80.2%, and a ROC-AUC of 87.7%. CNN-LSTM outperforms all models, achieving the highest accuracy, precision, and recall at 95.3%, 95.1% for F1, and 97.7% for ROC-AUC, respectively. Consequently, the overall effectiveness of deep learning exceeds that of ML algorithms on the imbalanced dataset with two distinct classes.

**Table 5:** The performance of models for imbalanced dataset with two classes.

Classifiers	Accuracy	Precision	Recall	F1	ROC-AUC
NB	75.2	76.2	75.2	73.7	85.5
LR	77.4	77.3	77.4	76.8	87.0
**LSVC**	**80.3**	**80.3**	**80.3**	**80.2**	**87.7**
MLP	79.1	79.2	79.1	79.1	86.2
SVM	78.4	78.2	78.4	78.1	87.1
DT	71.8	71.6	71.8	71.6	70.1
RF	78.7	78.5	78.7	78.4	86.6
**CNN-LSTM**	**95.3**	**95.3**	**95.3**	**95.1**	**97.7**

The results in bold indicate the highest accuracy and performance of the machine and deep learning models.

The results of model evaluations on a balanced dataset with two classes are presented in Table 6. As shown in this table, MLP exhibits exceptional performance, outperforming other ML methods with an accuracy of 93.0%, a recall of 93.2%, and an F1 of 96.3%. CNN-LSTM excels among all models, achieving the highest accuracy, recall, and F1, scoring 98.0%, 98.1%, and 99.7% for precision and ROC-AUC, sequentially. Consequently, the overall efficacy of deep learning surpasses that of ML algorithms on the balanced dataset with two distinct classes. Moreover, the outcomes demonstrate enhanced performance for all models while using the balanced dataset.

**Table 6:** The performance of models for a balanced dataset with two classes.

Classifiers	Accuracy	Precision	Recall	F1	ROC-AUC
NB	86.3	86.9	86.3	86.3	94.3
LR	88.1	88.4	88.1	88.0	95.6
LSVC	91.6	91.8	91.6	91.6	97.0
**MLP**	**93.0**	**93.2**	**93.0**	**93.0**	**96.3**
SVM	91.8	92.0	91.8	91.8	97.7
DT	88.0	88.3	88.0	88.0	88.0
RF	90.3	90.5	90.3	90.3	97.9
**CNN-LSTM**	**98.0**	**98.1**	**98.0**	**98.0**	**99.7**

The results in bold indicate the highest accuracy and performance of the machine and deep learning models.

Examining Tables 5 and 6 reveals that the models demonstrate improved performance in predicting two classes compared to four classes, in both cases of imbalanced and balanced datasets.

Additionally, two statistical tests, namely, the one-way ANOVA and Tukey's HSD test, were conducted to identify significant differences in the provided comparisons. The one-way ANOVA test aims to detect an overall significant difference between the results, while Tukey's HSD test facilitates pairwise comparisons to identify which specific pairs exhibit significant differences. According to the results of Tukey's HSD test, the MLP, SVM, LSVC, and CNN-LSTM methods demonstrate significantly better performance compared to the others. Specifically, as Table 7 reveals, Tukey's HSD test indicates a statistically significant difference in the performance of the CNN-LSTM method compared to all other methods.

**Table 7:** The results of Tukey's HSD test.

Pair	Difference	SE	Critical mean	*P* value
(CNN-LSTM) – LSVC	5.64	0.8737	3.5351	**.001629**
(CNN-LSTM) – MLP	4.66	0.8737	3.5351	**.008158**
(CNN-LSTM) – SVM	5.34	0.8737	3.5351	**.002662**
LSVC – MLP	0.98	0.8737	3.5351	.8565
LSVC – SVM	0.3	0.8737	3.5351	.9948

The results in bold indicate the highest accuracy and performance of the machine and deep learning models.

Nevertheless, the one-way ANOVA test reveals a significant difference across the results overall, suggesting that at least one of the methods exhibits statistically significant superiority.

Overall, we anticipated a significant contrast in performance between the CNN-LSTM model and traditional ML models. Traditional ML models typically treat text data as a bag of words or features, while CNN-LSTM models excel in capturing the sequential nature of text data. This capability enables them to potentially uncover deeper relationships and patterns within the text. It's crucial to emphasize that our model's training included embedding words into dense vector representations, which empowered it to effectively capture semantic similarities and distinctions. This methodology facilitated the generalization of learned patterns across different severity categories, thereby leading to better performance and enhancing the model's robustness.

## Discussion

There exists a considerable amount of research dedicated to exploring the application of NLP techniques within the healthcare domain.

In 2023, a systematic review was conducted to assess the utilization of NLP for extracting and retrieving information from clinical notes in dentistry across various databases. The review includes 17 studies, with 10 focusing on the development and evaluation of NLP methods, and 7 concentrating on NLP-based information retrieval in dental records. While there was observed improvement in reporting quality over time, many studies lacked crucial reproducibility details. Primary NLP methods included document classification and entity extraction, with some studies comparing NLP to non-NLP approaches. These findings underscore the necessity for standardized reporting and enhanced integration of NLP methods to optimize the utility of dental clinical notes in healthcare decision-making and population health endeavours.^10^

Sheikhalishahi et al. carried out a study on NLP methods in analysing clinical notes related to chronic diseases, focusing on the challenges faced. They identified 106 relevant articles out of 2652, covering 43 chronic diseases across 10 groups. While circulatory diseases were well-studied, endocrine and metabolic diseases were less explored due to differences in the data structure. The review highlighted a trend towards ML, especially in disease phenotype classification. However, challenges persist in understanding entity relations, temporal extraction, utilizing alternative clinical knowledge sources, and accessing large-scale, deidentified clinical data. Overall, the research emphasizes the necessity for further development and exploration to enhance NLP's capacity to analyse clinical narratives associated with chronic diseases.^11^

Another study aims to develop a method employing standardized vocabularies, clinical expertise, and NLP to extract symptom information from electronic health record notes. Five diverse symptom concepts were piloted using this method: constipation, depressed mood, disturbed sleep, fatigue, and palpitations. By expanding synonym lists through the Unified Medical Language System and clinical notes, the method significantly increased the number of synonym words or expressions for each symptom concept. NimbleMiner, an open-source NLP tool, was utilized for symptom identification, achieving excellent performance. This approach holds significant potential in facilitating symptom science research by accurately and efficiently extracting symptom information from electronic health record notes.^12^

Moreover, There exists a considerable amount of research concerning dental and oral issues. In clinical practice, the application of DL in CBCT can assist doctors in their diagnosis. This involves a range of preprocessing, segmentation, and classification techniques that form an automated dental identification system, facilitating the work of dentists.^13^^,^^14^ Most previous research has focused on using deep learning models to address oral and dental problems, particularly in analysing images.^15^^,^^16^

In 2023, Wenjie Fan and colleagues conducted a comprehensive review, which concentrated on analysing CBCT images and examining the correlation between teeth and surrounding tissues. They employed DL models to improve the accuracy and efficiency of their analysis. The review included an examination of various studies sourced from reputable scientific repositories such as PubMed, IEEE, Google Scholar, and Web of Science, covering research up to December 2022.^13^

In 2022, Kim et al. introduced an automatic tooth segmentation method based on CBCT imaging, simplifying the problem by breaking it down into smaller ones. Initially, they transformed the 3D image into a 2D representation and identified the 2D teeth. Subsequently, they captured loose and tight regions of interest. Finally, they segmented the accurate 3D tooth using these regions of interest. The accuracy achieved was up to 93.35%, with a Dice score of 94.79%.^13^^,^^17^ Many studies have looked into tooth segmentation and identification, and they all obtained great results.^18^, ^19^, ^20^, ^21^

An important aspect of our research focused on IE. An experiment performed in 2022 provided valuable insights into evaluating treatment quality through a sentiment analysis-based deep learning classification model. The study introduces a novel ML-based unsupervised sentiment analysis technique that integrates various deep learning methods, statistical approaches, and clustering. Experimental results demonstrate that the proposed method achieves 93% accuracy, outperforming other state-of-the-art approaches.^22^

We utilized a standard CNN-LSTM model to address our goal of estimating the severity of each patient's report. There is also an enhanced version of the CNN-LSTM model proposed by Deng et al.^23^ that incorporates BERT and an attention mechanism with CNN-LSTM to tackle the inefficiencies of current models in effectively handling long-term dependencies in natural language.^23^

Esmaeilyfard et al.^15^ evaluated deep learning algorithms for diagnosing and classifying tooth caries in CBCT images. Using a dataset containing carious and noncarious molar cases, they demonstrated that a multiple-input CNN achieved high diagnostic accuracy, sensitivity, specificity, and F1 score. The study concluded that deep learning, especially CNNs, holds promise for improving dental caries detection and classification, offering accessible diagnostic enhancements in dentistry, particularly in underserved areas.

Our research is distinctive in that we focus on examining and analysing the textual reports accompanying CBCT images in each patient's records. We compiled a comprehensive dataset, enriched with annotations from an expert, and subsequently extracted the text reports of each patient as our input data. This unique methodology sets our study apart from others in the field. While deep learning models are commonly used for predicting and classifying textual data in various domains such as Twitter data and COVID reports,^24^, ^25^, ^26^ studies employing CNN-LSTM on text reports of oral and dental patients are scarce.

Moreover, we carried out a comparative analysis at the end of our study to assess the performance of deep learning and traditional ML models. While many studies have utilized ML models to investigate the operation and classification of textual data, categorizing text in the context of health presents distinct challenges within text classification.

Several studies have explored the utilization of ML models in dentistry. ML studies published from 1 January 2015 to 31 May 2021, were scrutinized from diverse databases. Researchers analysed publication trends and ML tasks across clinical fields and assessed bias risk and reporting standards. The study revealed significant bias risk and moderate adherence to reporting standards, underscoring the importance of standardized outcome metrics for facilitating result comparisons.^27^

ML algorithms in NLP, such as Support Vector Machines and Latent Dirichlet Allocation, have shown effectiveness in tasks like classifying patient record notes.^28^^,^^29^

Sentiment analysis in ML primarily relies on supervised learning and ensemble techniques. In the supervised learning approach, a dataset containing labelled instances of tweets is utilized to train an ML model using classification algorithms like SVM, Bayesian classifier, and Entropy classifier.^30^, ^31^, ^32^, ^33^, ^34^ This training process classifies tweets into sentiment categories such as positive, neutral, and negative. Subsequently, the trained model is employed to predict the sentiment of new tweets. One notable drawback of the ML approach is its dependence on the creation of a substantial labelled training dataset, as the model's performance is directly influenced by the quality and quantity of the dataset.^35^^,^^36^

Another study investigated ML in tweet classification, proposing an ML algorithm based on optimization for categorizing tweets into different classes. Through three stages including data preprocessing, feature extraction using an optimization method, and updating a training set, the proposed algorithm achieved a maximum accuracy of 89.47% compared to other ML algorithms. Additionally, it proved to be faster and reduced overall data processing time, making it more suitable for larger datasets.^37^

Several studies have investigated the classification of tweets using the CNN-LSTM model, renowned for its efficacy in text data classification.^25^^,^^26^ This model combination, integrating CNNs and LSTM networks, has demonstrated notable success in various NLP tasks, including sentiment analysis and text classification.

Considering that certain regions lack access to dentists or during exceptional circumstances such as the pandemic, implementing this system proves immensely beneficial, particularly for high-risk patients. Utilizing CNN-LSTM can facilitate the classification of patients based on specific parameters. However, to ensure the precision of predictions, we enlisted the assistance of a dental professional to review the results.

Furthermore, employing CNN-LSTMs for the classification of dental issues necessitates precise data and the training of networks with extensive and diverse datasets containing ample records. While this method shows promise, it still requires further refinement and enhancement to optimize its effectiveness and accuracy in segregating high-risk patients from low-risk patients with dental and oral problems.

## Conclusion

In this study, we compiled an extensive set of CBCT radiography reports to distinguish emergency cases of dental and maxillofacial lesions from nonemergency ones. After construction of an annotated dataset (managed by three specialists), we proposed a CNN-LSTM model, specifically tailored with input parameters to address this challenge. The developed model was evaluated using 5-fold cross-validation, in two situations of imbalanced and balanced datasets. The results achieved by the proposed deep learning model were compared with some well-known ML models such as MNB, LR, LSVC, RF, MLP, DT, and SVM, as well. The findings indicated that CNN-LSTM outperforms other ML models in all datasets, highlighting the superior accuracy of the deep learning algorithm compared to traditional ML models. In conclusion, the results of the analysis, incorporating two tests (one-way ANOVA and Tukey's HSD), affirm the robust performance of CNN-LSTM.

To advance and enhance future research endeavours, the following actions can be considered:

• Exploring solutions to further enhance the accuracy of the models.
• Investigating the performance of the models on larger datasets to assess scalability.
• Incorporating additional features into the dataset for a more comprehensive analysis.

## CRediT authorship contribution statement

**Sare Mahdavifar:** Conceptualization, Methodology, Software, Investigation, Validation, Writing – original draft, Writing – review & editing. **Seyed Mostafa Fakhrahmad:** Conceptualization, Formal analysis, Supervision, Project administration, Funding acquisition, Writing – review & editing. **Elham Ansarifard:** Resources, Investigation, Data curation, Visualization, Writing – review & editing.

## Conflict of interest

The authors declare that they have no known competing financial interests or personal relationships that could have appeared to influence the work reported in this article.

## Footnotes

1 Convolutional neural network – long short-term memory.

2 Area under curve.

3 Receiver operating characteristic curve
